# Kidney care in patients with cancer: perspectives from the onconephrology committee of the Brazilian Society of Nephrology

**DOI:** 10.1590/1806-9282.2024S121

**Published:** 2024-06-07

**Authors:** Germana Alves de Brito, Renato Antunes Caires, Fernanda Oliveira Coelho, Mariana Fontes Turano Campos, Danielle Figueiredo da Cunha, Elerson Carlos Costalonga, Benedito Jorge Pereira, Marcelino de Souza Durão, Fernanda Amorim, Ana Maria Emrich dos Santos, Felipe Leite Guedes, Verônica Torres Costa e Silva

**Affiliations:** 1 A.C. Camargo Cancer Center, Nephrology Service – São Paulo (SP), Brazil.; 2 São Paulo State Cancer, Institute e University of São Paulo School of Medicine – São Paulo (SP), Brazil.; 3 San Rafael Hospital, Nephrology Service – Salvador (BA), Brazil.; 4 Private Practitioner – Rio de Janeiro (RJ), Brazil.; 5 National Cancer Institute, Nephrology Service – Rio de Janeiro (RJ), Brazil.; 6 Universidade Federal de São Paulo, Paulista School of Medicine, Discipline of Nephrology – São Paulo (SP), Brazil.; 7 Hospital Israelita Albert Einstein, Kidney Transplant Unit – São Paulo (SP), Brazil.; 8 State Public Servant Hospital, Nephrology Service – São Paulo (SP), Brazil.; 9 Londrina Cancer Hospital, Nephrology Service – Londrina (PR), Brazil.; 10 Universidade Federal do Rio Grande do Norte, Nephrology Service – Natal (RN), Brazil.; 11 Universidade de São Paulo, Faculty of Medicine, Medical Research Laboratory (LIM) 16 – São Paulo (SP), Brazil.

## INTRODUCTION

The number of new cases of cancer is expanding worldwide, with a projection of 28.4 million cases in 2040, an increase of 47% compared to 2020^1^. Conversely, global 5-year relative cancer survival has increased from 50% in 1975 to approximately 70% in 2014 due to early cancer diagnosis^2^. In consequence, there is an unprecedented population of cancer patients under treatment and follow-up in high-, middle-, and low-income regions.

Cancer incidence has been increasing in Latin American countries, such as Brazil. The Brazilian National Cancer Institute (INCA) estimates 704,000 new cancer cases per year for the period 2023–2025^3^. In Brazil, cancer diagnosis and treatment are usually concentrated in tertiary centers located in large metropolitan areas, which are able to provide access to essential cancer treatments such as surgery, standard chemotherapy, radiotherapy, and palliative care. For instance, the south and southeast regions, which have better socioeconomic status, account for about 70% of cancer incidence due to greater access to cancer screening exams and more robust cancer registries compared to lower-income areas of the country^3^. Despite local disparities, Brazil has shown a significant improvement in the quality and availability of data about cancer, an expansion in the number of cancer centers, and a substantial improvement in anticancer treatment.

## INTERPLAY BETWEEN CANCER AND KIDNEY CARE: MAIN ASPECTS OF KIDNEY CARE IN PATIENTS WITH CANCER

The rapid advancement of new therapies in recent years has substantially enhanced the survival prospects of cancer patients, prompting a multifaceted intersection between nephrology and oncology. Nephrologists are increasingly encountering different aspects of this interrelation, such as patients with chronic kidney disease (CKD), kidney transplant recipients who are diagnosed with cancer, and patients who may also experience acute kidney injury (AKI), electrolyte imbalances, and proteinuria. These manifestations can arise from the cancer itself or the therapies employed (Table 1)^4^.

**Table 1:** Clinical issues related to kidney care in cancer patients.

Acute kidney injury
Volume depletion: vomiting, diarrhea
Sepsis and septic shock
Surgery-related kidney injury
Severe electrolyte disorders
Hyponatremia
Hypercalcemia
Hypo/hyperkalemia
Renal toxicity of non-chemotherapeutic drug treatments
Analgesics
Opioids
Antibiotics
Biphosphonates
Pump proton inhibitors
Onco-urology
Obstructive uropathy
Post-nephrectomy AKI and CKD
Radiofrequency and cryoablation of kidney mass
Post-cystectomy kidney dysfunction
Post-TURP syndrome
Management of CKD patients
Use of ESA
Management of hypertension
GFR estimation: equations based on SCr vs. measured GFR through exogenous markers
Modifications of dosing of chemotherapy in patients with CKD and ESRD
Issues related to kidney transplantation
Assessment of cancer risk and cancer screening strategies in transplanted patients
Management of immunosuppression in transplanted patients with cancer
Transplantation in cancer patients after cancer treatment
Management of proteinuria
Kidney infiltration by cancer disease
Tumor or tumor treatment-related microangiopathies and GN
Radiotherapy-induced kidney injury
Management of radiotherapy in ESRD patients
Tumor lysis syndrome
Myeloma-related kidney injury

AKI: acute kidney injury; CKD: chronic kidney disease; TURP: transurethral resection of the prostate; ESA: erythropoiesis stimulating agents; GFR: glomerular filtration rate; SCr: serum creatinine level; ESRD: end-stage renal disease; GN: glomerulonephritis.

### Acute kidney injury in patients with cancer

Acute kidney injury is a common complication in cancer patients, and its incidence is dependent on the type of cancer, ongoing treatment, and patient's comorbidities or clinical events, ranging from 11 to 22%. Patients in intensive care units (ICUs) are at a higher risk of AKI, as are those undergoing hematopoietic stem cell transplantation (HSCT). Some specific points in the evaluation of patients with AKI and cancer include:

1. Diagnosis of high-risk neoplasms for AKI such as symptomatic multiple myeloma, bladder cancer, and leukemia^5^.
2. Evaluate the risk of obstructive uropathy or local complications associated with the cancer.
3. Assess the risk of tumor lysis syndrome, an urgent medical situation due to the potential for life-threatening arrhythmias, respiratory failure, and AKI^6^.
4. Consider any prior medical procedures that might have reduced renal mass, such as nephrectomies or other ablative treatments.
5. Review the patient's history of exposure to chemotherapy agents and other therapies. Conventional chemotherapies still play a central role in the treatment of many cancer types and are known to promote vascular, glomerular, and tubular injuries^6^. Cisplatin is associated with a significant incidence of AKI, reported in 32% of adults receiving a single dose^7^. Immune checkpoint inhibitors (ICPis) enhance the activity and proliferation of cytotoxic T cells, leading to acute tubulointerstitial nephritis in most AKI cases^8^. AKI occurs in 19–24% of patients treated by CAR-T cell therapies^9^.
6. Check for the use of nononcologic medications with nephrotoxic potential, like nonsteroidal anti-inflammatory drugs (NSAIDs), antibiotics, and proton pump inhibitors (PPIs)^10^.

### Electrolyte disorders in patients with cancer

Among patients diagnosed with cancer, hyponatremia stands out as the most prevalent electrolyte disturbance, and there is a clear relationship between serum sodium levels, hospital stay, and mortality^11^. Syndrome of inappropriate antidiuretic hormone (SIADH) secretion is the leading cause of hyponatremia in this population, and when directly linked to cancer, it often results from increased ADH production due to primary or metastatic neoplasms affecting the lungs or brain. Decreased oral intake and gastrointestinal losses in those patients can also contribute to hyponatremia, hypernatremia, hypomagnesemia, hypokalemia, and hypophosphatemia^12^. Hypercalcemia is also a common electrolyte complication seen in cancer patients, occurring in up to 30% of cases. It is essential to emphasize that successful treatment of these conditions hinges on addressing the cancer itself^13^. The renal effects of chemotherapy and targeted therapies, including their direct tubular effects, can induce losses of potassium, magnesium, and phosphate in the kidneys. Therefore, it is imperative to monitor these ions during therapy^14^.

### Chronic kidney disease and cancer

Chronic kidney disease is common in patients with cancer, with more than half of patients with solid tumors reportedly having an estimated glomerular filtration rate (GFR) (eGFR) of less than 90 mL/min/1.73 m^2^. Most of these patients have stage 2 (eGFR, 60–89 mL/min) or 3 (eGFR, 30–59 mL/min) (40–50% and 15–20%, respectively) CKD^15^.

Chemotherapy medications are often metabolized by the kidneys, making the accurate estimation of GFR one of the primary challenges in the kidney care of these patients. It is essential to determine the proper dosage of medications, including chemotherapy, in order to avoid excessive kidney and systemic toxicity or underdosing and compromise of cancer outcomes^14^.

Other clinically relevant aspects of routine care for CKD patients, such as the management of anemia, treatment of hypertension, diabetes, and mineral bone disease, should be tailored to the cancer scenario (Table 1). Patients considered to be cured, without active cancer disease, and with expected survival long enough to benefit from specific interventions should be treated in a similar manner as CKD patients without cancer. These patients are suitable for more intensive blood pressure control, glycemic levels, and strategies to prevent CKD progression. Conversely, patients with reduced survival should be managed, prioritizing control of symptoms and prevention of acute complications such as hyperkalemia, acidosis, and congestion.

### Transplantation and oncology

Kidney transplantation offers a new lease on life for individuals with end-stage renal disease. However, it also introduces unique challenges, particularly concerning cancer risk. Transplant recipients are at an increased risk of developing certain cancers due to long-term immunosuppressive therapy. As a result, regular screenings to detect cancer at an early, treatable stage and vigilant monitoring for post-transplant lymphoproliferative disorder (PTLD) and other hematologic malignancies are part of the interplay between nephrologists and oncologists^16^.

The timing of transplantation in patients with a prior history of malignancy remains another topic of considerable debate. With the ongoing rapid advancements in cancer treatments, transplant centers should revisit their existing protocols for establishing pretransplant waiting periods for cancer-free status^17^.

On the other hand, when cancer is diagnosed in kidney transplant recipients, the challenge lies in finding a balance between managing cancer effectively and preserving the transplanted kidney. Additionally, immunosuppressive regimens may need to be modified or temporarily withdrawn during cancer treatment^16^.

## ONCONEPHROLOGY GLOBALLY

Thanks to a worldwide effort, led mainly by centers in North America and Europe, a considerable amount of information has been accumulated in the last few years in the onconephrology scenario. Several initiatives in assistance, education, and research areas have been developed worldwide, which are crucial to moving this field forward.

### Onconephrology fellowships

One of the most critical initiatives in the field was the development of training programs. Currently, there are six onconephrology fellowship programs in North America—five in the United States (US) and one in Canada^18^. The programs include outpatient clinics and inpatient rotations in the hematology, oncology, palliative care, and oncopharmacology groups, as well as research activities, highlighting the close working relationship between oncologists, hematologists, and nephrologists as part of a multidisciplinary team^19^. Although onconephrology fellowships are still lacking in South America and other regions of the globe, the inclusion of onconephrology rotations and a dedicated education program have been included as core curriculum in general nephrology fellowships, such as the Nephrology Fellowship Program from the University of São Paulo School of Medicine in Brazil.

### Onconephrology centers

One crucial step in this field was the development of well-designed onconephrology centers and clinics, such as the one led by M. Gallieni and collaborators in Milan^20^. The working team includes nephrologists, hematologists, oncologists, a dedicated data manager, and a nursing care coordinator with specialized training in onconephrology. The assistance is grounded in multidisciplinary meetings incorporating urologists, radiation therapists, pathologists, radiologists, palliative care providers, and others for case management and guided by dedicated protocols and a set of performance indicators. Since onconephrology is presently more experience-based than evidence-based, these centers are successful models of addressing onconephrological issues in a dedicated setting with a tight interspecialty collaboration, able to provide better kidney care for patients with cancer.

### Onconephrology societies and working groups

In the onconephrology field, a number of medical societies and working groups have been created in recent years. The most robust was the American Society of Onconephrology (ASON), launched in the US in 2021, including nephrologists from multiple countries worldwide. Since its creation, ASON has established a partnership with the International Society of Nephrology, leading to active collaborations with nephrology societies across the globe, including Asia, Africa, and South America. ASON has created several research groups aiming to enhance scientific collaboration among its members and developed an agenda of research topics, apart from a relevant role in spreading scientific content (papers, talks, and podcasts) through its communication channels (website, Twitter account, and Instagram) to educate the nephrology community on onconephrology topics. Scientific discussions, webinars, and scientific meetings are freely advertised. ASON is also working on position statement documents to offer guidance to health professionals on critical aspects related to the kidney care of patients with cancer and has developed an initiative to support cataloging the renal toxicities of anticancer therapies^21^.

The increase in onconephrology research and education initiatives is also observed in Europe. The Onconephrology Research Group of French Speaking Language has developed several activities and published many papers^22^. In Spain, a working group called “onconephrology” has been created by the Spanish Society of Nephrology. The working group holds regular meetings, and multicenter collaborative projects are proposed in different hospitals with onconephrology units, combined with training courses for nephrologists^23^. The Portuguese Society of Nephrology has created a similar working group^24^. As mentioned earlier, Galliani's group has been a leading team in onconephrology research and assistance in Italy.

### Journals, meetings, and other initiatives

The number of papers published in high-impact journals addressing topics in the onconephrology field has dramatically increased, with some special editions in a few over the years. In 2017, the Journal of Onconephrology, the first peer-reviewed periodic exclusively devoted to the topic, was released and is now a major reference for onconephrologists^25^. Every year, an international symposium on onconephrology, now organized by the ASON leadership, takes place in academic institutions in the US, gathering the foremost researchers at a global level. In parallel, symposiums, premeetings, and scientific and poster sections are now routine at the American Society of Nephrology meetings, the World Congress of Nephrology, and other top nephrology congresses worldwide.

Despite these exciting and unprecedented steps on a global level, onconephrology is still an incipient field in Latin American countries such as Brazil, which face local challenges in the kidney care of patients with cancer.

## CHALLENGES AND PRIORITY INITIATIVES FROM THE ONCONEPHROLOGY COMMITTEE OF THE BRAZILIAN SOCIETY OF NEPHROLOGY TO IMPROVE KIDNEY CARE IN FOR PATIENTS WITH CANCER IN BRAZIL

Brazil is a country of continental dimension, with a population of 220 million people, in which 90% of cancer treatment relies on the public health system, struggling with financial limitations to fund appropriate care and research. Additionally, cancer treatment is concentrated in large cancer centers located in major cities, especially in the south and southeast regions, where nephrology training usually occurs, contributing to geographic disparities in kidney care.

The increasing number of cancer patients under treatment in the country is leading to an unprecedented and unmet demand for nephrologists with experience and training in onconephrology in order to appropriately treat patients with cancer and kidney diseases. Unfortunately, the opportunities available for onconephrology education and research in Brazil are scarce. There are only a few onconephrology groups working in cancer centers; there are no onconephrology fellowship programs; rotations in onconephrology during nephrology fellowships are rare; and research projects in the field are still incipient. To face these challenges, the Brazilian Society of Nephrology (SBN) officially created an onconephrology committee with the mission of developing initiatives to promote onconephrology education and research in the country. The committee gathers nephrologists with expertise in the care of cancer patients. This is the first working group devoted to the onconephrology field attached to a medical or nephrology association in Latin America, and the committee is currently organizing the first onconephrology premeeting within an SBN Congress in 2024 and is working in partnership with the ASON to collaborate in scientific activities.

**Figure 1:**
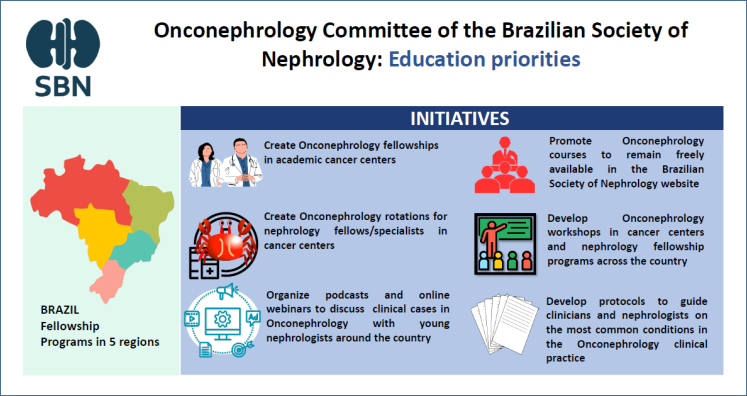
Education initiatives undertaken by the onconephrology committee of the Brazilian Society of Nephrology.

The committee has identified six main education initiatives to improve the background and training of nephrologists in the onconephrology field (Figure 1): (1) Create onconephrology fellowships in large academic cancer centers grounded in a multidisciplinary collaboration with oncology, hematology, and urology services. These specialized doctors could work in sequence in smaller cities and rural areas in order to replicate their experience and educate doctors working locally. (2) Create onconephrology rotations in cancer services during nephrology and internal medicine fellowships across the country, which could be an effective entry for young doctors into the field. (3) Organize podcasts and online webinars to promote the discussion of clinical cases led by members of the committee with young nephrologists. These discussions will be advertised and supported by the SBN, available free of charge to the medical community, and may count on the collaboration of hematologists, oncologists, urologists, and nephrologists from cancer centers across the country and from the ASON. (4) Promote onconephrology courses that cover the most frequent topics in clinical practice, such as multiple myeloma, obstructive uropathy, the toxicity of new anticancer treatments, palliative care, and kidney transplant patients with cancer, on the SBN platform. Talks and audiovisual material should be free of charge and remain available online for consultation. (5) Develop onconephrology workshops in cancer centers and nephrology fellowship programs nationwide to discuss common topics in onconephrology clinical practice, participate in clinical rounds, and debate cases in clinical meetings. (6) Develop protocols that can guide clinicians and nephrologists on the most common questions and conditions related to the nephrology practice during the patient's journey with cancer in order to standardize routine practices.

**Figure 2:**
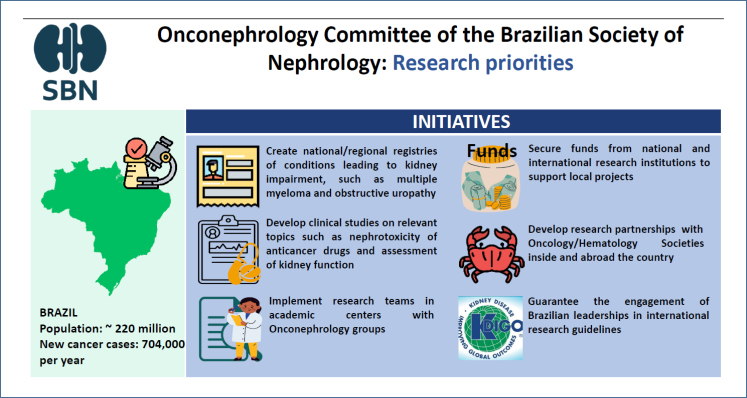
Research initiatives suggested by the onconephrology committee of the Brazilian Society of Nephrology.

The committee also elected six actions to stimulate research (Figure 2): (1) Create national and regional registries of common conditions leading to kidney impairment, such as multiple myeloma and obstructive uropathy, which suffer from a paucity of data. Accurate information on the incidence and prognosis of these diseases could provide valuable information to plan better management and tailored treatment strategies that could improve patients’ prognosis. These data could also inform health policies by government agencies. (2) Develop clinical studies on relevant topics to our population, such as the nephrotoxicity of anticancer drugs and the assessment of kidney function. Most of the studies in the onconephrology field are developed in high-income countries, including North American and European patients, assessing interventions (e.g., expensive chemotherapy drugs) that are frequently unavailable in Brazil. Consequently, there is a paucity of clinical data to respond to local needs that can be applied to our distinct population profile. National clinical studies are fundamental to mitigating this drawback. (3) Implement research teams in Brazilian academic centers with onconephrology groups aiming to fund, train, and organize research teams capable of conducting well-designed projects. Research structure is still immature in a subspecialty as incipient as onconephrology. Well-established scientific groups can take the lead, gather graduate students from different geographic areas, educate junior investigators, consolidate expertise areas, and allow for increased funding. (4) Secure funds from national and international research institutions to support local projects that can provide funds to overcome local budget constraints. There is a shortage of funds for studies not sponsored by the pharmaceutical industry. Therefore, local research largely depends on public funding from government agencies. Partnerships with national and international private, nonprofit, or public research institutions can fill this gap. (5) Develop research partnerships with oncology and hematology societies inside and outside the country in order to share the same research and logistics structure, optimizing resources and enabling a multidisciplinary approach. (6) Guarantee the engagement of Brazilian leadership in international research guidelines, increasing the representation of these leaders in global discussions and guidelines.

The committee's main objectives are to minimize inequalities, train young nephrologists, and contribute to the improvement of onconephrology research in Brazil. Our efforts aim to involve onconephrology groups throughout the country to meet local needs and promote the establishment and growth of onconephrology as the new subspecialty in the war against kidney diseases. We hope these initiatives will improve kidney care, leading to better quality of life and the survival of cancer patients in Brazil.
